# Epidermal growth factor receptor‑targeted antibody nimotuzumab combined with chemoradiotherapy improves survival in patients with locally advanced head and neck squamous cell carcinoma: a propensity score matching real‐world study

**DOI:** 10.1002/mco2.608

**Published:** 2024-07-02

**Authors:** Peng Zhang, Xinxin Zhang, Jinyi Lang, Shaoxiong Wu, Yan Sun, Peiguo Wang, Sufang Qiu, Xiaodong Huang, Guoxin Ren, Kun Liu, Xiaojing Du, Shaowen Xiao, Zhongqiu Wang, Youliang Weng, Ye Zhang, Hang Zhou, Wenyong Tu, Chenping Zhang, Junlin Yi

**Affiliations:** ^1^ Department of Radiation Oncology, Radiation Oncology Key Laboratory of Sichuan Province, Sichuan Clinical Research Center for Cancer Sichuan Cancer Hospital & Institute, Sichuan Cancer Center, Affiliated Cancer Hospital of University of Electronic Science and Technology of China Chengdu China; ^2^ Senior Department of Otolaryngology‐Head & Neck Surgery the Sixth Medical Center of PLA General Hospital, National Clinical Research Center for Otolaryngologic Diseases Beijing China; ^3^ Department of Radiation Oncology, State Key Laboratory of Oncology in South China Sun Yat‐sen University Cancer Center Guangzhou China; ^4^ Department of Radiation Oncology Beijing Cancer Hospital Beijing China; ^5^ Department of Radiation Oncology Tianjin Medical University Cancer Institute & Hospital Tianjin China; ^6^ Department of Radiation Head and Neck Oncology Fujian Cancer Hospital Fuzhou China; ^7^ Department of Radiation Oncology National Cancer Center/National Clinical Research Center for Cancer/Cancer Hospital, Chinese Academy of Medical Sciences and Peking Union Medical College Beijing China; ^8^ Department of Oral and Maxillofacial Tumor Surgery Shanghai Ninth People's Hospital, Shanghai Jiaotong University School of Medicine Shanghai China

**Keywords:** chemoradiotherapy, IMRT, LA‐HNSCC, nimotuzumab, safety, survival benefits

## Abstract

Patients with locally advanced head and neck squamous cell carcinoma (LA‐HNSCC) have poor survival outcomes. The real‐world efficacy of nimotuzumab plus intensity modulated radiotherapy (IMRT)‐based chemoradiotherapy in patients with LA‐HNSCC remains unclear. A total of 25,442 HNSCC patients were screened, and 612 patients were matched by propensity score matching (PSM) (1:1). PSM was utilized to balance known confounding factors. Patients who completed at least five doses of nimotuzumab were identified as study group. The primary end point was 3‐year overall survival (OS) rate. Log‐rank test examined the difference between two survival curves and Cloglog transformation test was performed to compare survival at a fixed time point. The median follow‐up time was 54.2 (95% confidence interval [CI]: 52.7–55.9) months. The study group was associated with improved OS (hazard ratio [HR] = 0.75, 95% CI: 0.57–0.99, *p* = 0.038) and progression‐free survival (PFS) (HR = 0.74, 95% CI: 0.58–0.96, *p* = 0.021). Subgroup analysis revealed that aged 50–60 year, IV, N2, radiotherapy dose ≥ 60 Gy, without previous surgery, and neoadjuvant therapy have a trend of survival benefit with nimotuzumab. Nimotuzumab showed favorable safety, only 0.2% had nimotuzumab‐related severe adverse events. Our study indicated the nimotuzumab plus chemoradiotherapy provides survival benefits and safety for LA‐HNSCC patients in an IMRT era.

## Graphical abstract

Nimotuzumab plus chemoradiotherapy provided manageable safety and survival benefits with improved OS and PFS for LA‐HNSCC patients in an IMRT era. And subgroup analysis revealed that patients with aged 50–60 year, stage IV, N2, radiotherapy dose ≥60 Gy, without previous surgery, and neoadjuvant therapy have a trend of survival benefit with nimotuzumab.

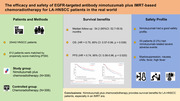

## 1 INTRODUCTION

Head and neck cancer (HNC) is the eighth most common cancer worldwide, with 830,000 new individuals each year, and the eighth highest cause of mortality among all malignancies, with 430,000 new deaths globally per year.^1^ Head and neck squamous cell carcinoma (HNSCC) accounts for 90% of all HNCs,^2^, ^3^ originating from the epithelial cells of the mucosal linings in the oral cavity, larynx, and pharynx.^4^, ^5^ HNSCC is generally diagnosed in the older population,^6^ and its primary risk factors of HNSCC are smoking and alcoholism.^7^ More than half of the new cases are locally advanced HNSCC (LA‐HNSCC) at diagnosis.^8^ Previous study revealed that chemoradiotherapy is the most common treatment for LA‐HNSCC, but the long‐term survival rate and quality of life of patients after treatment are unsatisfactory.^9^ Furthermore, despite the significant advances in conventional therapies such as surgery, radiotherapy, and chemotherapy, the prognosis of patients remains poor. Approximately 25% of patients will develop a second primary tumor within five years of diagnosis,^10^, ^11^ and the 5‐year overall survival (OS) rate of patients with LA‐HNSCC is less than 40%.^12^ As a result, new and safe treatments are required to improve the survival of patients with LA‐HNSCC.

Epidermal growth factor receptor (EGFR) is a transmembrane glycoprotein receptor encoded by chromosomal proto‐oncogenes,^13^, ^14^ and belongs to the transmembrane receptor tyrosine kinase family.^15^ EGFR is involved in the regulation of cell growth, migration, differentiation, and metabolism.^16^ Numerous findings proved that overexpression of EGFR significantly correlated with initiation and progression.^17^, ^18^ EGFR pathway also plays an essential role in multiple tumor types, including breast, colorectal, cervical, and lung cancer.^19^, ^20^ Moreover, the overexpression of EGFR indicates the enhanced resistance of tumors to radiotherapy and chemotherapy, which is a key predictive factor and therapeutic target for poor prognosis of cancers.^21^ In particular, EGFR is upregulated in up to 90% of HNSCC, and its oncogenic stimulus is associated with the biological activities of overexpression, amplification, and activation of HNSCC.^22^, ^23^ Thus, the anti‐EGFR monoclonal antibodies (mAbs) may benefit LA‐HNSCC patients.

Nimotuzumab (h‐R3), a humanized mAb, has been approved for HNC in 24 countries worldwide. It was developed in Cuba that targets the human EGFR, inhibits the EGFR‐dependent signaling through binding to the EGFR extracellular domain.^24^, ^25^ The monoclonal has an important antitumor activity, mainly through influencing the biological process of cell proliferation, growth, and angiogenesis,^26^ also enhancing the sensitivity of radiotherapy.^27^ Compared with other antibodies, the clinical use of nimotuzumab lacks serious skin‐toxicity adverse effects.^28^ Nimotuzumab has been approved in several countries, for various cancers such as head and neck, pancreatic, esophageal, glioma, and non‐small cell lung cancer.^29^ The clinical benefits of nimotuzumab in the treatment of HNSCC have been demonstrated with various studies. Recently a randomized phase III trial from India, nimotuzumab plus conventional radiotherapy based concurrent chemoradiotherapy (CCRT) provided survival benefits for patients with LA‐HNSCC.^30^ The median progression‐free survival (PFS) of patients with and without nimotuzumab were 60.3 and 21 months, respectively (*p* = 0.023). The 2y‐PFS was significantly higher (61.8 vs. 50.1%, *p* = 0.0044), and 2y‐OS tended to be higher (63.8 vs. 57.7%, *p* = 0.16). However, it lacks real‐world study in an intensity modulated radiotherapy (IMRT) era to verify the efficacy of nimotuzumab plus chemoradiotherapy, especially in China. As an EGFR‐targeted drug, nimotuzumab has been shown to improve survival in HNSCC in retrospective small‐sample studies, and a phase III randomized controlled study from India indicated that it can improve PFS, but there was no statistical difference in OS.^30^ In the real‐world, whether the benefit of nimotuzumab regimen could be repeated is unclear. So it is necessary to design a real‐world study to investigate the survival benefit and safety of nimotuzumab combined with chemoradiotherapy.

We conducted a multicenter, retrospective, and controlled real‐world study to investigate whether nimotuzumab combined with chemoradiotherapy could improve survival in Chinese patients with LA‐HNSCC, especially in an IMRT era.

## 2 RESULTS

### 2.1 Participant characteristics

**FIGURE 1:**
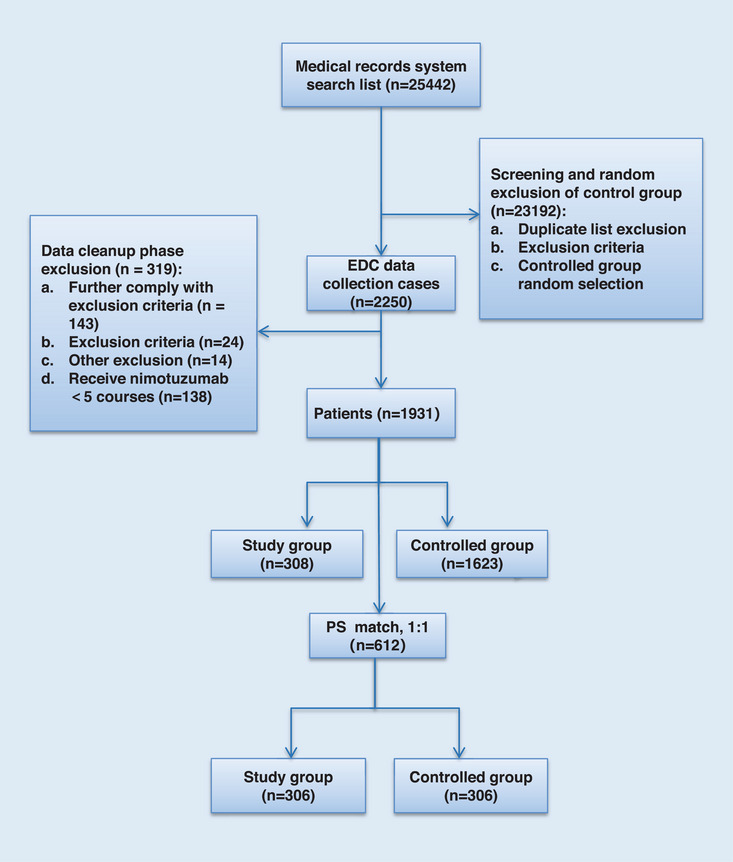
The flow chart of trial design and patients screening. EDC, electronic data capture; PS, propensity score.

First, 25,442 patients with HNSCC regardless of treatment were collected and further screened according to the inclusion and exclusion criteria. A total of 1931 patients were eligible, including 308 patients in the study group and 1623 patients in the controlled group. After propensity score matching (PSM), 612 patients (306 patients in each group) were included in the final analysis (Figure 1). The covariates for estimating PSM contains gender, age, overall stage, cancer type, chemotherapy cycle, radiotherapy dose, antitumor surgery, and neoadjuvant chemotherapy. The percentage of stage IV disease was approximately 80% in both groups. There were 107 (35.0%) patients in the study group and 88 (28.8%) patients in the controlled group who had not received previous surgery. The use of CCRT was 62.4% in the study group and 65.4% in the controlled group. A total of 250 (81.7%) patients received IMRT in the study group and 275 (89.8%) in the controlled group. The demographic characteristics are shown in Table 1.

**TABLE 1:** Baseline patient characteristics.

	No. (%)		
Characteristic	Controlled group (*N* = 306)	Study group (*N* = 306)	Standardized difference (%)
Gender			0.9
Male	255 (83.3)	256 (83.7)	
Female	51 (16.7)	50 (16.3)	
Age, mean (SD)	58.7 (11.30)	59.0 (11.44)	2.4
Nationality			3.4
The Han nationality	293 (95.8)	295 (96.4)	
Other	13 (4.2)	11 (3.6)	
BMI, mean (SD)	22.67 (3.109)	23.26 (3.460)	17.9
Smoking history			8.6
Yes	175 (60.3)	189 (64.5)	
No	115 (39.7)	104 (35.5)	
Drinking history			17.0
Yes	159 (54.5)	184 (62.8)	
No	133 (45.5)	109 (37.2)	
Drug allergy history			21.2
Yes	17 (5.6)	35 (11.4)	
No	289 (94.4)	271 (88.6)	
Without previous surgery	88 (28.8)	107 (35.0)	13.4
Classification of cancer			
Laryngeal carcinoma	40 (13.1)	42 (13.7)	1.9
Oral carcinoma	76 (24.8)	62 (20.3)	11.0
Oropharyngeal carcinoma	93 (30.4)	97 (31.7)	2.8
Hypopharyngeal carcinoma	97 (31.7)	105 (34.3)	5.6
Pathological type			
Squamous carcinoma	306 (100.0)	306 (100.0)	NA
Differentiation			
Undifferentiated	1 (0.5)	0 (0.0)	10.1
Poorly differentiated	39 (19.7)	35 (17.9)	4.5
Medium differentiation	68 (34.3)	79 (40.5)	12.8
Highly differentiated	32 (16.2)	24 (12.3)	11.0
Other	58 (29.3)	57 (29.2)	0.1
T stage			
T1	31 (10.1)	19 (6.2)	14.4
T2	82 (26.8)	78 (25.5)	3.0
T3	67 (21.9)	74 (24.2)	5.4
T4	125 (40.8)	132 (43.1)	4.6
N stage			
N0	43 (14.1)	41 (13.4)	1.9
N1	64 (20.9)	59 (19.3)	4.1
N2	173 (56.5)	193 (63.1)	13.4
N3	24 (7.8)	12 (3.9)	16.7
M stage			
M0	306 (100.0)	306 (100.0)	NA
Clinical stage			1.6
III	63 (20.6)	65 (21.2)	
IV	243 (79.4)	241 (78.8)	
Radiotherapy technology			
Intensity modulated radiotherapy (IMRT)	275 (89.9)	250 (81.7)	23.6
Three‐dimensional conformal radiotherapy (D‐CRT)	1 (0.3)	0 (0.0)	8.1
Others	30 (9.8)	56 (18.3)	24.6
Concurrent chemoradiotherapy (CCRT)	200 (65.4)	191 (62.4)	6.1
Neoadjuvant therapy (induction chemotherapy)	122 (39.9)	138 (45.1)	10.6
ECOG PS score			
0	27 (45.0)	34 (51.5)	13.1
1	31 (51.7)	28 (42.4)	18.6
2	2 (3.3)	3 (4.5)	6.2
3	0 (0.0)	1 (1.5)	17.5

### 2.2 Treatment

The dosage of nimotuzumab was 200 mg per week, the average total dose was 1373.1 ± 601.32 mg. The average radiotherapy dose was 60 Gy. The chemotherapy regimen was mainly platinum based.

### 2.3 Efficacy evaluation

**FIGURE 2:**
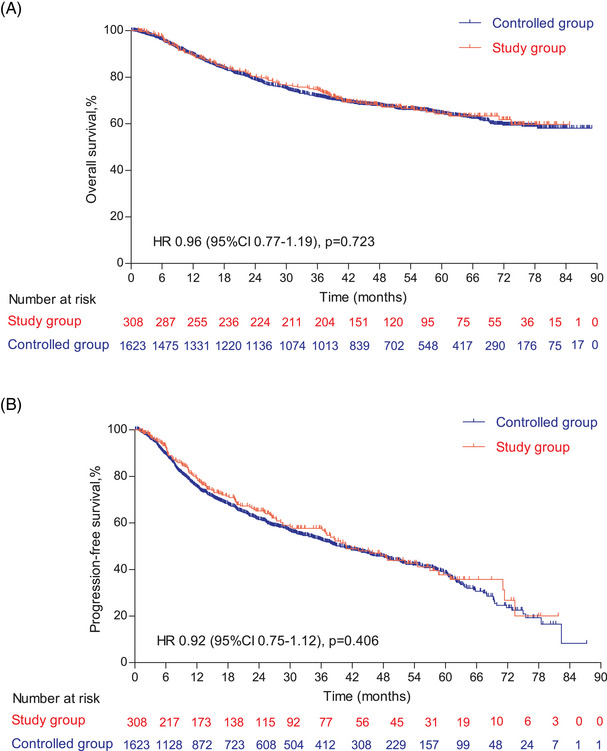
Survival outcomes in the controlled and the study group before propensity score matching (PSM). (A) The Kaplan–Meier curve of overall survival (OS) before PSM. (B) The Kaplan–Meier curve of progression‐free survival (PFS) before PSM.

**FIGURE 3:**
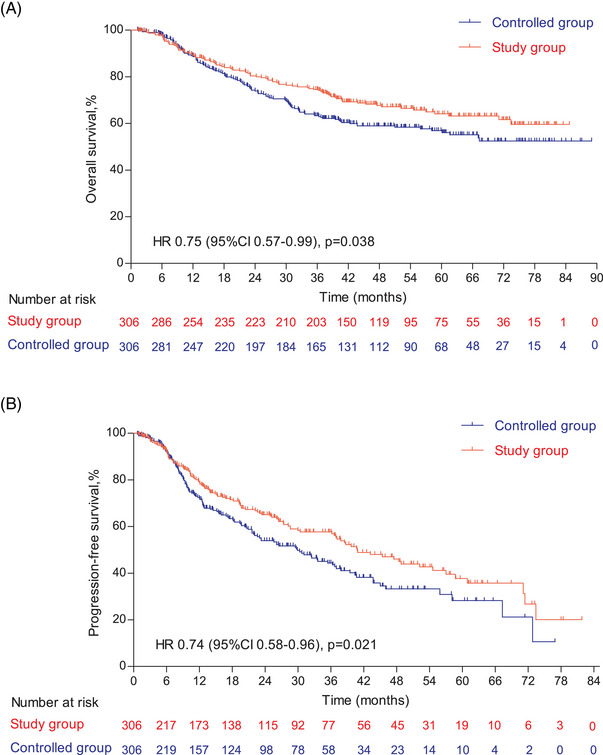
Survival outcomes in the controlled and the study group after propensity score matching (PSM). (A) The Kaplan–Meier curve of overall survival (OS) after PSM. (B) The Kaplan–Meier curve of progression‐free survival (PFS) after PSM.

**FIGURE 4:**
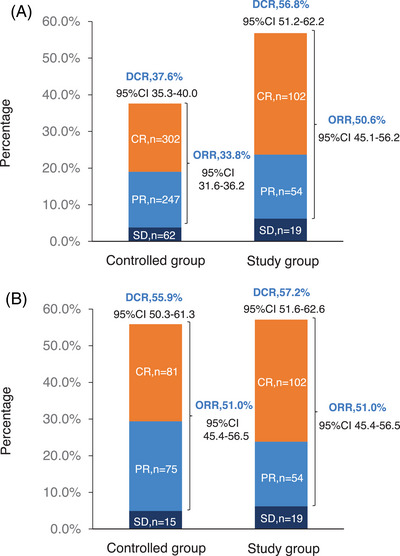
Tumor response in the controlled and the study group. (A) The objective response rate (ORR) and disease control rate (DCR) before propensity score matching (PSM). (B) The ORR and DCR after PSM.

The follow‐up was cut off on January 19, 2023 and the median follow‐up time was 54.2 (95% confidence interval [CI]: 52.7–55.9) months. Among the entire cohort, before PSM, the results indicated that there was no statistically significant difference in OS (HR = 0.96, 95% CI: 0.77–1.19, Log‐rank *p* = 0.723; 3y‐OS: 74.7 vs. 71.7%, Cloglog *p* = 0.316), PFS (HR = 0.92, 95% CI: 0.75–1.12, Log‐rank *p* = 0.406; 3y‐PFS: 57.7 vs. 52.7%, Cloglog *p* = 0.206). The objective response rate (ORR) and disease control rate (DCR) were 50.6 versus 33.8% (*p* < 0.001) and 56.8 versus 37.6% (*p* < 0.001), respectively. After PSM, the addition of nimotuzumab was associated with improved OS (HR = 0.75, 95% CI: 0.57–0.99, Log‐rank *p* = 0.038; 3y‐OS: 74.6 vs. 63.3%, Cloglog *p* = 0.004) as well as PFS (HR = 0.74, 95% CI: 0.58–0.96, Log‐rank *p* = 0.021; 3y‐PFS: 57.7 vs. 44.3%, Cloglog *p* = 0.009), and the study exhibited ORR (51.0 vs. 51.0%, Log rank *p* = 1.00) and DCR (57.2 vs. 55.9%, Log rank *p* = 0.744) with no statistical significance between the two groups (Figures 2, 3, 4).

**FIGURE 5:**
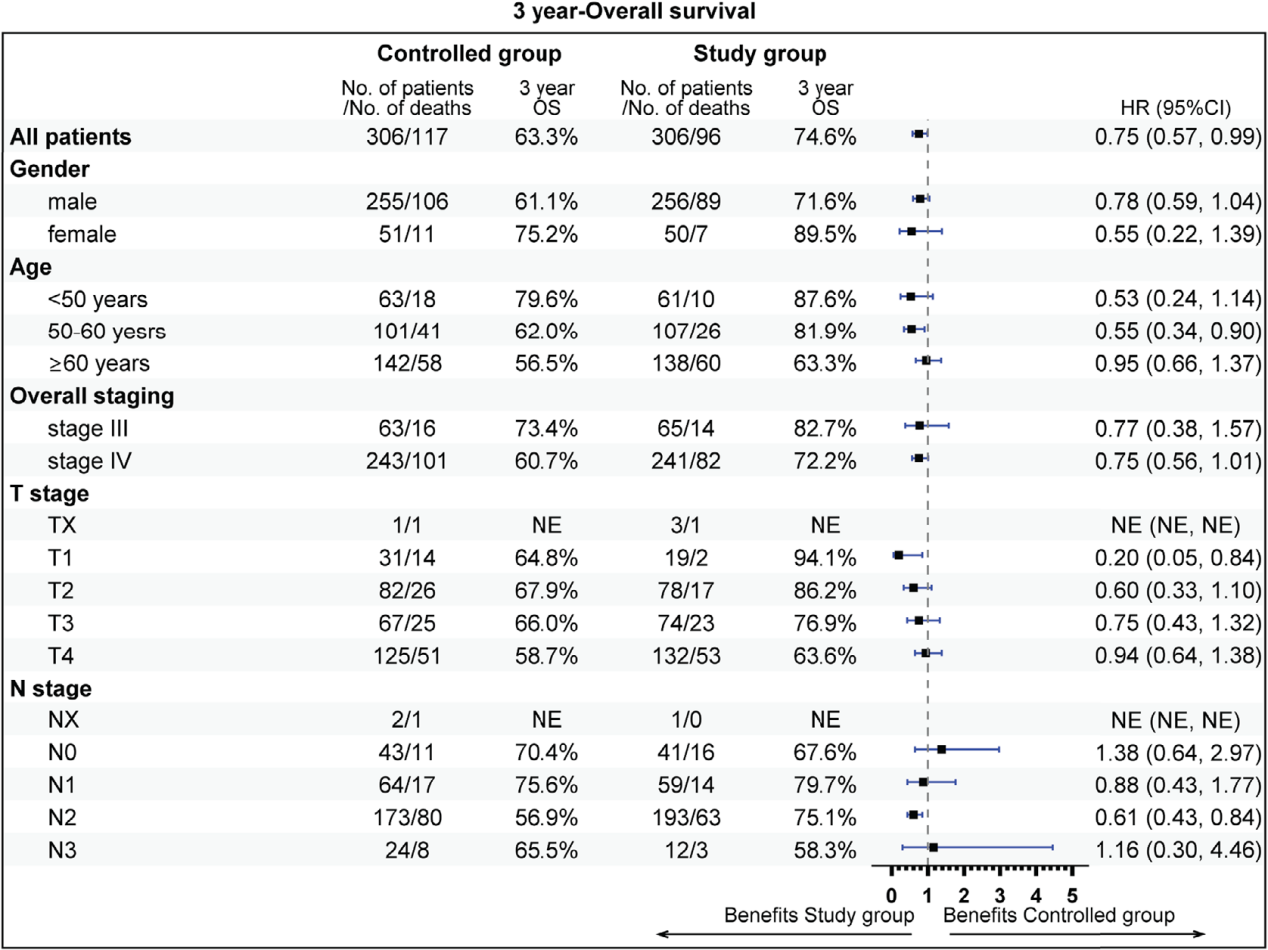
Subgroup analysis of overall survival (OS), the clinical variables includes gender, age, overall staging, T stage, and N stage.

**FIGURE 6:**
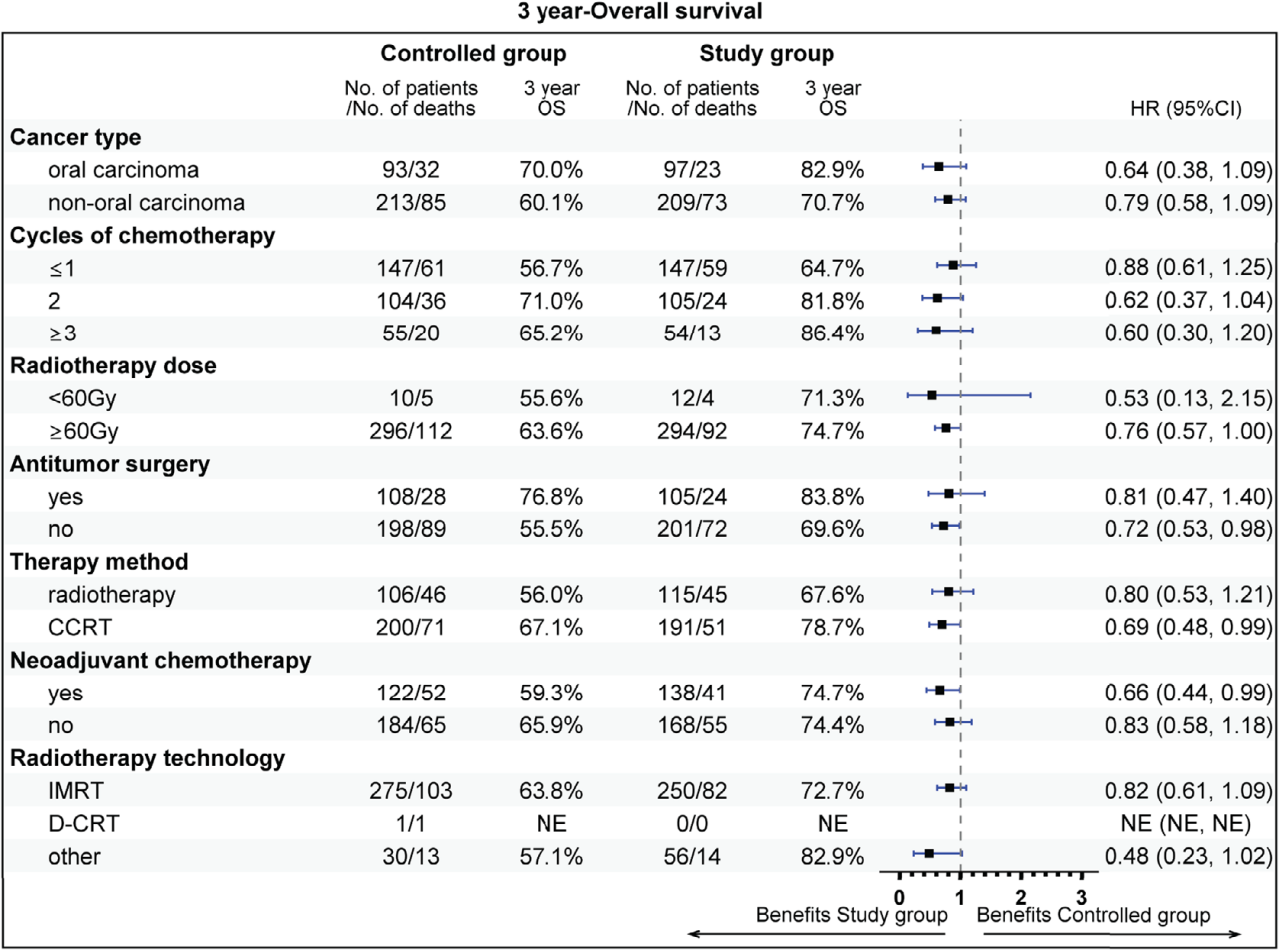
Subgroup analysis of overall survival (OS), the clinical variables includes cancer type, cycles of chemotherapy, radiotherapy dose, antitumor surgery, therapy method, neoadjuvant chemotherapy, and radiotherapy technology.

**FIGURE 7:**
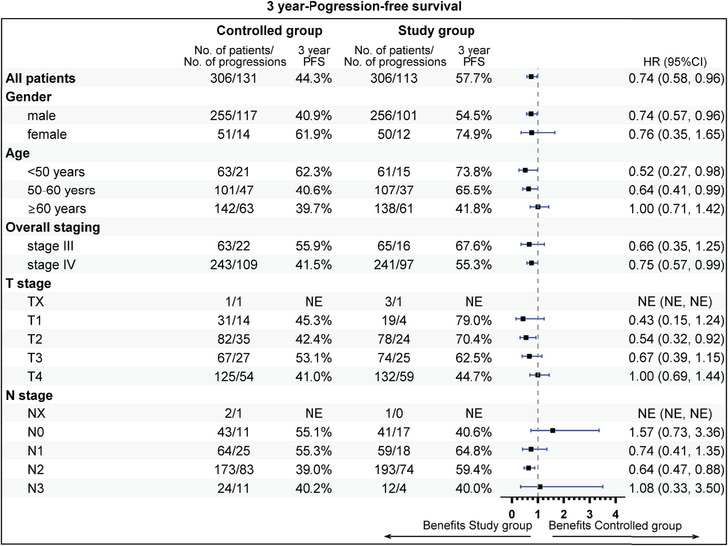
Subgroup analysis of progression‐free survival (PFS), the clinical variables includes gender, age, overall staging, T stage, and N stage.

**FIGURE 8:**
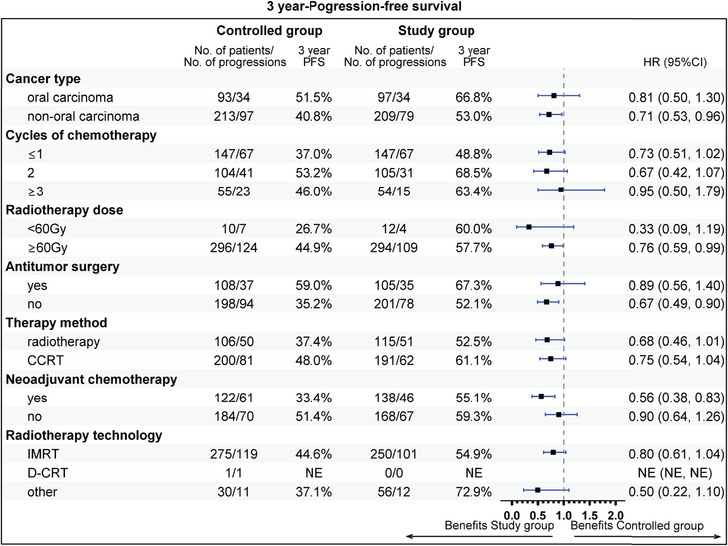
Subgroup analysis of progression‐free survival (PFS), the clinical variables includes cancer type, cycles of chemotherapy, radiotherapy dose, antitumor surgery, therapy method, neoadjuvant chemotherapy, and radiotherapy technology.

The subgroup analysis revealed that aged 50−60 years (81.9 vs. 62.0%), stage IV (72.2 vs. 60.7%), T1 stage (94.1 vs. 64.8%), N2 (75.1 vs. 56.9%), radiotherapy dose ≥60 Gy (74.7 vs. 63.6%), without previous surgery (69.6 vs. 55.5%), CCRT sub‐cohort (78.7 vs. 67.1%), and neoadjuvant therapy (74.7 vs. 59.3%) could gain more 3‐year OS rate benefit from the addition of nimotuzumab. Beneficial subgroup for 3‐year PFS rate included male (54.5 vs. 40.9%), age (<50 years, 73.8 vs. 62.3%; 50−60 years, 65.5 vs. 40.6%), stage IV (55.3 vs. 41.4%), T2 (70.4 vs. 42.4%), N2 (59.4 vs. 39.0%), nonoropharyngeal cancer (53.0 vs. 40.8%), radiotherapy dose ≥60 Gy (57.7 vs. 44.9%), without previous surgery (52.1 vs. 35.2%), and neoadjuvant therapy (55.1 vs. 33.3%) (Figures 5, 6, 7, 8).

### 2.4 Safety

According to the postmarketing data, from January 1, 2015 to December 31, 2018, a total of 24,976 patients received nimotuzumab, and it showed that nimotuzumab had a good safety profile. A total of 151 (0.6%) cases of postmarketing adverse reactions were reported, and 146 (0.6%) cases were nimotuzumab‐related. Fifty‐three patients (0.2%) had nimotuzumab‐related severe adverse events, mainly myelosuppression, nausea, chills, fever, high fever, etc. Previous studies indicated that chemoradiotherapy alone resulted in more grades 3−4 adverse reactions for HNC compared with nimotuzumab plus chemoradiotherapy,^31^ thus nimotuzumab demonstrating acceptable and manageable toxicity.

## 3 DISCUSSION

This multicenter, retrospective, real‐world research demonstrated that nimotuzumab combined with chemoradiotherapy significantly improved OS for patients with LA‐HNSCC, especially for those aged 50−60 years, stage IV, or N2 disease, nonoropharyngeal cancer, without previous surgery, radiotherapy dose ≥60 Gy, and neoadjuvant therapy. Furthermore, nimotuzumab demonstrated a good safety profile, which represented an underlying novel choice for LA‐HNSCC patients.

A previously published phase IIb research showed that nimotuzumab provides a long‐term survival benefit to patients with inoperable advanced HNSCC. In this study, 92 patients with advanced unresectable HNSCC were randomly divided into four groups: concurrent chemoradiotherapy + nimotuzumab group (CRT + Nimo group), concurrent chemoradiotherapy group (CRT group), concurrent radiotherapy + nimotuzumab group (RT + Nimo group), and concurrent radiotherapy group (RT group). The results indicated patients treated with RT + Nimo had a 24% reduction in the risk of death compared with those who received RT alone. Additionally, the median OS of patients treated with nimotuzumab was 49.38 months compared with 16.36 months in patients treated without nimotuzumab (*p* = 0.012). The risk of death in patients with nimotuzumab was 48% lower than that in patients without nimotuzumab, and the safety was good.^32^ The randomized and prospective phase III trial conducted in India demonstrated that nimotuzumab could prolong the survival time for LA‐HNSCC patients. In this research, 536 patients with stage III or IV LAHNSCC were eligible and classified into two treatment arms: cisplatin‐radiation arm (CRT) (n = 268) and nimotuzumab plus cisplatin‐radiation arm (NCRT) (*n* = 268). The 2y‐PFS was significantly elevated (61.8 vs. 50.1%, *p* = 0.0044), and the median PFS of patients with and without nimotuzumab was 60.3 months and 21 months (*p* = 0.023), respectively. The 2y‐OS tended to increase (63.8 vs. 57.7%, *p* = 0.16), but it was not statistically significant.^30^ Notably, our findings exhibited significantly elevated 3y‐PFS and advantageous 3y‐OS. After PSM the ORR and DCR were 51.0 versus 51.0% (*p* = 1.00) and 57.2 versus 55.9% (*p* = 0.744), respectively. And there was no statistically significant difference in OS and PFS before PSM. Additionally, above 85% patients received conventional radiotherapy in India study, whereas 81% patients received IMRT in this study. Therefore, our study uncovered that nimotuzumab plus IMRT and chemotherapy could significantly improve the survival of Chinese patients with LA‐HNSCC. Although the nimotuzumab plus chemoradiotherapy showed prolonged PFS and OS, there were no significant differences in ORR and DCR between the two treatment methods, so as in the Indian phase III trial.

Regarding safety, our study showed no serious adverse events with the addition of nimotuzumab, which is similar to the findings of previous studies indicating a low toxicity profile of nimotuzumab.^33^, ^34^ Systemic diseases, administration site reactions, and gastrointestinal diseases were the main adverse events in our research. Among these, and consistent with previous publications, the most common adverse reactions to nimotuzumab were: bone marrow suppression,^35^, ^36^ nausea,^37^, ^38^ chills,^39^ and fever.^40^ Anti‐EGFR antibodies generally have substantial skin toxicity, and the incidence rate is about 60−80%.^41^ In the randomized phase III trial (RTOG0522), the patients with stage III or IV HNC were classified into cetuximab (a mouse chimeric anti‐EGFR mAb) combined with cisplatin‐radiation group and cisplatin‐radiation alone group. We found that cetuximab plus cisplatin‐radiation has more obvious toxicity than cisplatin‐radiation alone, including a higher incidence of grades 3−4 radiation mucositis (43.2 vs. 33.3%) and rash.^42^ Meanwhile, the data confirmed by Bonner et al.^43^ indicated that adding cetuximab in radiotherapy exhibited apparent toxicity for patients with locoregionally advanced HNSCC, especially rash, with the rash of grades 3−5 accounting for 17%. However, only very few patients developed rashes in our trial, demonstrating that nimotuzumab, a humanized anti‐EGFR mAb, is much safer and has no added toxicity. In addition, evidence has determined that the stable attachment of nimotuzumab requires bivalent binding, so nimotuzumab selectively binds to tumor cells expressing moderate to high EGFR levels, thereby protecting healthy cells with low levels of EGFR, and thus severe skin toxicity does not occur.^32^, ^44^ Consequently, nimotuzumab combined with chemoradiotherapy is safe in the treatment of LA‐HNSCC.

There were some limitations in this study. First, as a retrospective study, there were many missing imaging data in this real‐world study, resulting in a high censoring rate, so the 3‐year locoregional control could not be obtained. A prospective study is needed for further verification. In addition, the testing for EGFR expression and human papillomavirus status was not performed in this research.

Our study is a real‐world study. Whether the findings of this research can be applicable to people in other countries would necessitate a big international multi‐center phase III randomized controlled trial.

In conclusion, nimotuzumab combined with chemoradiotherapy indicated improved survival with the acceptable and manageable toxicity profile compared with chemoradiotherapy alone. Moreover, it showed OS benefit, and the patient aged 50−60 years, stage IV, N2 disease, without previous surgery, radiotherapy dose ≥60 Gy, and neoadjuvant therapy may indicate a trend of survival benefit, especially in an IMRT era.

## 4 METHODS

### 4.1 Trial design and patients

All HNSCC patients treated in eight hospitals in different regions of China between January 2015 and December 2018 were retrospectively screened from medical records system. The eight centers included Chinese Academy of Medical Sciences and Peking Union Medical College, Sichuan Cancer Hospital & Institute, Shanghai Ninth People's Hospital, The Sixth Medical Center of PLA General Hospital, Sun Yat‐sen University Cancer Center, Beijing Cancer Hospital, Tianjin Medical University Cancer Institute & Hospital, and Fujian Cancer Hospital. Eligible patients were aged ≥18 years with unlimited gender, Eastern Cooperative Oncology Group Performance Status (ECOG PS) score of 0−3, and had stages III–IVb of HNSCC (including oral, oropharyngeal, hypopharyngeal, and laryngeal, but not nasopharyngeal cancers) confirmed by histopathology or cytology. Patients who received nimotuzumab plus chemoradiotherapy were considered study group, and those who did not receive nimotuzumab were considered controlled group. We excluded patients with multiple primary malignant tumors other than head and neck (except cured basal cell carcinoma of the skin and carcinoma in situ of the cervix) or those treated with other targeted therapy, immunotherapy, or antitumor medicine.

First, PSM was implemented by logistic regression. Second, the inverse probability treatment weighting was performed. For PSM, the study group and the controlled group were matched with a 1:1 ratio of nearest neighbor matching, and the caliper value was 0.2 times the standard deviation of PS logit. Additionally, the PSM, as a statistical method removing confounding bias from observational cohorts with impossible benefit of randomization, worked by the nearest neighbor matching method, and each patient in the study group was systematically matched with three patients sharing the closet propensity score from the controlled group. After PSM (1:1), all patients in two groups were matched, and we confirmed there were not any statistical differences in patient baseline characteristics with minimal confounding bias in properly determining treatment effect. The known confounding factors, such as clinical stage (AJCC, 7 ed.), primary location and so on, were screened by directed acyclic graph and incorporated into PSM matching process as matching factors. Sensitivity analysis was performed for all matching factors in order to resolve bias.

Totally 25,442 patients in the medical records system were screened, among which 23,192 patients did not meet the inclusion criteria or met the exclusion criteria after preliminary screening and were not collected into the electronic data capture (EDC) system. Finally, 2250 patients were collected into the EDC system. After data cleaning, 319 patients were excluded, Among them, 143 patients did not meet the inclusion criteria, 24 patients met the exclusion criteria, 138 patients were excluded due to receiving nimotuzumab <5 courses during radiotherapy/chemotherapy, 14 patients were excluded in other situations. So 1931 patients were included in the matching model. There were 308 in the study group and 1623 in the controlled group. Based on PSM (1:1), there were 612 patients, including 306 in the study group and 306 in the controlled group.

This study was carried out by following the principles of the Declaration of Helsinki and Good Clinical Practice guidelines. It was also approved by the ethics committees of the 8 participating hospitals in China.

### 4.2 Treatment procedures and end points

In this retrospective trial, patients completed at least five doses of nimotuzumab combined with chemoradiotherapy or chemoradiotherapy alone, with follow‐up data ending on January 19, 2023. The primary end point was 3 year‐OS rate. OS was defined as the time from the start of treatment to death. The secondary end points were 3 year‐PFS rate, ORR, and DCR. PFS was considered the time from the start of treatment to the disease progression or death from any cause. ORR was defined as the proportion of patients who achieved a prespecified reduction in tumor size and maintained the minimum duration. Finally, DCR was considered the percentage of patients who achieved remission or stable disease (SD) after treatment.

The National Cancer Institute Common Terminology Criteria for Adverse Events (version 3.0) was implemented to assess the adverse events in this trial. It was planned to collect Adverse Drug Reaction (ADR) in China during the study period (including but not limited to clinical trials, head and neck tumors) from the National ADR Monitoring Center feedback and the postmarket report of Biotech Pharmaceutical Co., Ltd.

In addition, the Response Evaluation Criteria in Solid Tumors (version 1.1) was applied to determine the imaging results, and according to that, the response status was considered as: complete response, partial response, SD, and progressive disease.

### 4.3 Statistical analysis

The sampling estimation was evaluated through test efficiency and model robustness. In terms of test efficiency, the primary end point was OS. According to retrospective studies,^8^, ^45^, ^46^ it was assumed that the 3‐year OS rate in the controlled group will be 50%, which increased to 62% after adding nimotuzumab. The primary analysis was set to adopt 1:1 PSM with a two‐sided test level of *α* = 0.05, and *β* = 0.10. The survival module in the PASS15 software was used to obtain a sample size of 266 cases in each group. As an alternative hypothesis, the proportion of successful PSM was 70%, and the sample size of each group was 380 cases. Conservatively estimating a 20% of the cases with incomplete information, the sample size required for each group was 475 cases. Assuming that the number of cases collected in the controlled group will be three times that of the study group, the required sample size was 1900 cases (475 cases in the study group and 1425 cases in the controlled group). Regarding model robustness, the OS for the study and controlled groups was expected to be approximately 56%. Assuming that there were 15 variables included in the model and the events per variable were set to 30, 450 deaths will be needed. So, the effective sample size of the two groups had to be at least 1023 cases. Conservatively estimating a 20% of the cases with incomplete information, the planned sample size was 1280 (320 in the study group and 960 in the controlled group). Taken together, the sample size was determined to be at least 1900 cases (475 cases in the study group and 1425 cases in the controlled group).

The binary unconditioned logistic regression was applied to generate the PS model. The grouping was presented as the dependent variable and the covariate was primarily determined based on clinical significance and exploratory analysis. The patients were matched based on PS after logit transformation. The matching method adopted 1:1 greedy matching algorithm, and the standard deviation of PS after logit transformation was set to 0.2 times of the caliper value. Based on the matched data set, the matched variables were statistically described, and the standard difference was calculated with the limit of 10% as the equilibrium effect. The accuracy and rationality of PS model were depicted in a residual graph. Kaplan–Meier curve was performed to calculate OS and PFS, and the nonstratified Log‐rank test was applied to calculate the *p* value. Also, the nonstratified Cox proportional hazard model was used to estimate the HR and the 95% CI. The ORR and DCR were calculated by two classification indicators. The Pearson chi square (*χ*2) test was implemented for comparison between groups and *p* ≤ 0.05 was considered statistically significant. SAS software (version 9.4) was used to perform all statistical analyses.

*Elimination of bias by PSM*: Based on Directed Acyclic Graph combined with Backdoor Criterion, model convergence and clinical importance determine covariates included into the PS model. The matching method was performed by the nearest and caliper matching as the main analysis. Baseline variables with large standardized differences after PSM were corrected by the intensity of association with grouping and outcome (*E*‐value) not reaching 1.74 or less than 0.57 (1/1.74) at the same time.

## AUTHOR CONTRIBUTIONS

Junlin Yi, Jinyi Lang, Chenping Zhang, Peng Zhang, and Xinxin Zhang designed and drafted the work. Xinxin Zhang, Shaoxiong Wu, Yan Sun, Peiguo Wang, Sufang Qiu, Xiaodong Huang, Ye Zhang, Peng Zhang, Hang Zhou, Guoxin Ren, Wenyong Tu, Kun Liu, Xiaojing Du, Shaowen Xiao, Zhongqiu Wang, and Youliang Weng acquired the data. Junlin Yi, Jinyi Lang, Chenping Zhang, and Xinxin Zhang analyzed the data. Junlin Yi substantively revised the work. All authors read and approved the final version.

## CONFLICT OF INTEREST STATEMENT

The authors declare no potential conflict of interest. Biotech Pharmaceutical Co., Ltd. as a member of study designer funded this trial, without involving in the data collection, analysis, interpretation, and manuscript writing processes.

## ETHICS STATEMENT

It was approved by the Ethics Committee of National Cancer Center/Cancer Hospital, Chinese Academy of Medical Sciences, and Peking Union Medical College with APPROVAL NUMBER (Approval No.: 21/306‐2977) and Registration NMUBER (Clinicaltrials.gov Identifier: NCT04949503).

## Data Availability

The data generated in this study are available upon request from the corresponding author.
